# Anesthetic Agents of Plant Origin: A Review of Phytochemicals with Anesthetic Activity

**DOI:** 10.3390/molecules22081369

**Published:** 2017-08-18

**Authors:** Hironori Tsuchiya

**Affiliations:** Department of Dental Basic Education, Asahi University School of Dentistry, 1851 Hozumi, Mizuho, Gifu 501-0296, Japan; hiro@dent.asahi-u.ac.jp; Tel.: +81-58-329-1266

**Keywords:** phytochemical, local anesthetic, general anesthetic, plant origin, pharmacological mechanism, lead compound

## Abstract

The majority of currently used anesthetic agents are derived from or associated with natural products, especially plants, as evidenced by cocaine that was isolated from coca (*Erythroxylum coca*, Erythroxylaceae) and became a prototype of modern local anesthetics and by thymol and eugenol contained in thyme (*Thymus vulgaris*, Lamiaceae) and clove (*Syzygium aromaticum*, Myrtaceae), respectively, both of which are structurally and mechanistically similar to intravenous phenolic anesthetics. This paper reviews different classes of phytochemicals with the anesthetic activity and their characteristic molecular structures that could be lead compounds for anesthetics and anesthesia-related drugs. Phytochemicals in research papers published between 1996 and 2016 were retrieved from the point of view of well-known modes of anesthetic action, that is, the mechanistic interactions with Na^+^ channels, γ-aminobutyric acid type A receptors, *N*-methyl-d-aspartate receptors and lipid membranes. The searched phytochemicals include terpenoids, alkaloids and flavonoids because they have been frequently reported to possess local anesthetic, general anesthetic, antinociceptive, analgesic or sedative property. Clinical applicability of phytochemicals to local and general anesthesia is discussed by referring to animal in vivo experiments and human pre-clinical trials. This review will give structural suggestions for novel anesthetic agents of plant origin.

## 1. Introduction

Many of the currently used medicines originate from natural products, especially plants. Drugs and plants are closely related to each other through the use of traditional medicines or ethnomedicines that are mainly prepared from plants [1]. Representative drugs of plant origin include the anticholinergic atropine (from *Atropa belladonna*, Solanaceae), the antimalarial quinine (from *Cinchona officinalis*, Rubiaceae), the cardiotonic digitoxin (from *Digitalis purpurea*, Plantaginaceae), the antitussive codeine (from *Papaver somniferum*, Papaveraceae), and the analgesic salicylate and its derivative aspirin (from *Salix alba*, Salicaceae). Plants and herbs are the sources of not only crude drugs, but also bioactive compounds that could lead to novel drug structures [2].

Because medicinal plants and herbs have been used since ancient times for relieving pain caused by disease, injury and surgery, some of them contributed to the development of modern anesthesia [3]. Cocaine, the first local anesthetic, originates from a specific plant alkaloid and the widely used intravenous anesthetic propofol shares the partial structure and pharmacological mechanism with certain plant terpenoids. The anesthetic adjunct morphine and the injectable muscle relaxant *d*-tubocurarine were also derived from opium poppy and the arrow poison curare prepared with vine plants, respectively.

For discovering drug candidates, plants of interest are screened for the presence of bioactive components, phytochemicals responsible for the bioactivity are isolated, their molecular structures are identified, and then the original structures of phytochemicals may be semi-synthetically modified to enhance the activity or reduce the toxicity [4]. In particular, a research strategy based on the pharmacological mechanism is very effective to obtain phytochemical lead compounds for anesthetics and anesthesia-related drugs [5].

This paper reviews different classes of phytochemicals with the significant anesthetic activity and their characteristic molecular structures from the point of view of well-known modes of anesthetic action, that is, the mechanistic interactions with ion channels, receptors and lipid membranes. The review focuses on terpenoids, alkaloids and flavonoids because they have been frequently reported to possess local anesthetic, general anesthetic, antinociceptive, analgesic or sedative property. Besides these phytochemicals, plants are able to produce anesthetic ethylene and vinyl ether especially under stress [6]. Although these stress hormones were previously applied to inhalational anesthesia [7], they are infrequently used today due to toxicity and degradation during storage. Therefore, such phytochemical alkenes and ethers are not included in this review. Clinical applicability and implication of the relevant terpenoids, alkaloids and flavonoids are discussed by referring to animal in vivo experiments and human pre-clinical trials with them.

## 2. Method

The present review is based on published articles and information retrieved from PubMed/MEDLINE, ACS Publications and Google Scholar. Databases were searched from 1996 to 2016. The relevant research papers published in recognized international journals and on-line journals in English were preferred, but review articles of specific importance were also included, although non-English language citations were excluded. Published case reports and abstracts were used when their complete articles were not available. The searches were carried out by using the following terms or combinations thereof: “local anesthetic”, “general anesthetic”, “antinociceptive”, “analgesic” and “sedative” for pharmacological activity; “plant component”, “terpenoid”, “alkaloid” and “flavonoid” for phytochemicals; and “Na^+^ channel”, “γ-aminobutyric acid type A (GABA_A_) receptor”, “*N*-methyl-d-aspartate (NMDA) receptor” and “membrane interaction” for molecular mechanisms. Collected articles were reviewed by title, abstract and text for relevance, with preference to more recent publications. Their bibliographies were also searched for additional references.

## 3. Plants That Contributed to the Development of Anesthesia

### 3.1. Local Anesthetics Derived from Plant Alkaloid

**Figure 1:**
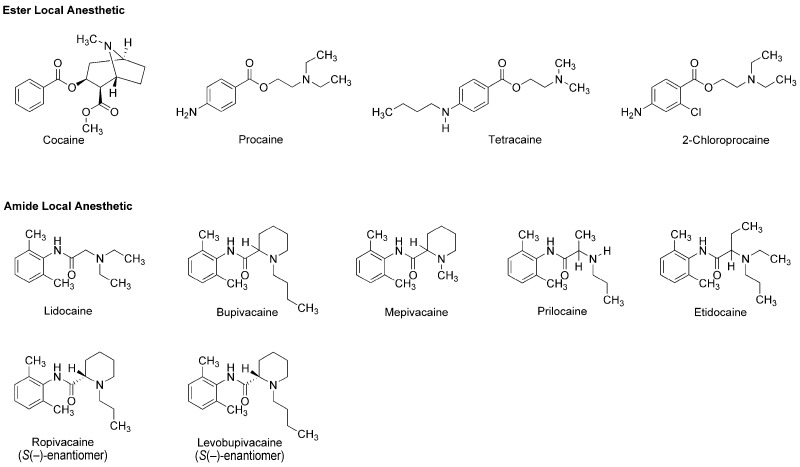
Local anesthetics derived from the plant alkaloid cocaine.

The earliest use and cultivation of coca, a shrub of the genus *Erythroxylum*, in the Andean region of South America, are very likely to date back to several thousand years B.C. [8]. However, a pain-relieving substance had been neither structurally identified nor clinically applied until the leaves of *Erythroxylum coca* (Erythroxylaceae) were taken from Peru to Europe. In 1860, Albert Niemann in Göttingen reported that chewing coca leaves took away tongue feeling and taste. He isolated an active substance from them and named it as “cocaine”. Sigmund Freud, the founder of psychoanalysis, read Niemann’s report and considered using cocaine to overcome morphine addiction. He encouraged his colleague Carl Koller, a resident in ophthalmology in Vienna, to take part in the study of cocaine. Koller experimented on animals and humans by dropping a cocaine solution onto the eyeballs and found the numbing effect of cocaine on the eye. Shortly thereafter, he successfully performed cataract surgery using cocaine as a topical anesthetic in 1884 [9]. After his first clinical introduction, modern local anesthesia began with the development of injectable cocaine and its use for spinal anesthesia. In 1898, Richard Willstätter structurally identified cocaine as methyl (1*R*,2*R*,3*S*,5*S*)-3-(benzoyloxy)-8-methyl-8-azabicyclo[3.2.1]octane-2-carboxylate (Figure 1). However, as undesirable (toxicity and addiction) and problematic (short duration and difficult sterilization) properties of cocaine became apparent, its molecular structure was modified to obtain safer and more effective drugs. In 1904, Alfred Einhorn synthesized procaine to replace cocaine, followed by a series of synthetic drugs: tetracaine, lidocaine, 2-chloroprocaine, bupivacaine, mepivacaine, prilocaine, etidocaine, ropivacaine and levobupivacaine (Figure 1). Local anesthetics derived from plant alkaloid cocaine, especially amide-type drugs, have been successfully used in a wide range of situations to relieve and control pain. The most widely recognized mode of action for local anesthetics is the interaction with voltage-gated Na^+^ channels to inhibit sensory and motor functions [10]. Anesthetic molecules penetrate through the lipid barriers of nerve sheaths and diffuse across the lipid bilayers of cell membranes so that they access the intracellular or cell-interior binding sites on Na^+^ channels embedded in membranes. Local anesthetics also diffuse into lipid bilayers and act on membrane-constituting lipids, modifying the physicochemical properties of neuronal and cardiomyocyte membranes [11].

### 3.2. General Anesthetics Associated with Plant Terpenoids

**Figure 2:**
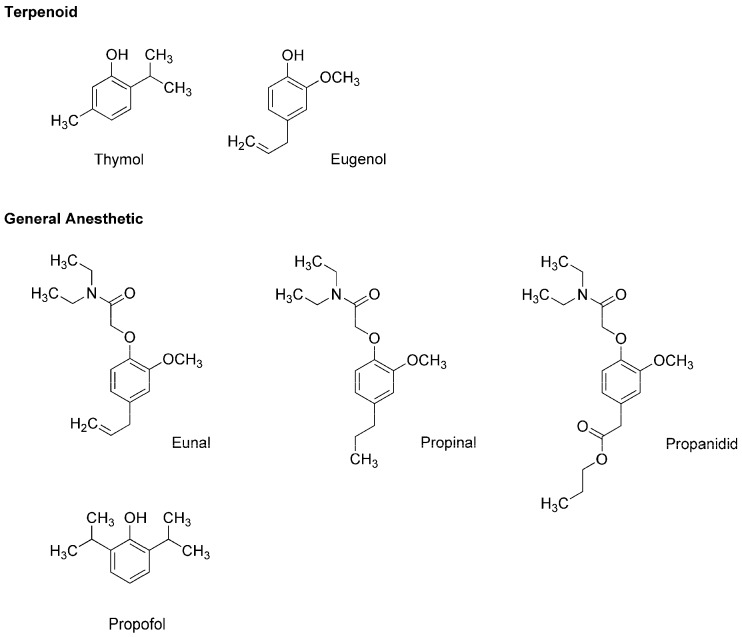
General anesthetics associated with plant terpenoid thymol and eugenol.

Thymol (2-isopropyl-5-methylphenol) and eugenol (4-allyl-2-methoxyphenol) (Figure 2) are contained in thyme (*Thymus vulgaris*, Lamiaceae) and clove (*Syzygium aromaticum*, Myrtaceae), respectively, both of which are well-known for culinary and medicinal uses. Eugenol also occurs in herbs such as nutmeg (*Myristica fragrans*, Myristicaceae), cinnamon (*Cinnamomum verum*, Lauraceae) and basil (*Ocimum basilicum*, Lamiaceae). Because these terpenoid phenols possess antinociceptive and antibacterial effects, they have been extensively used in dental practice as a sedative or analgesic agent for pulpitis, toothache and dental hyperalgesia. In addition, thymol, eugenol and their structurally-related compounds exhibit general anesthetic activity.

In the 1950s, the utility as a general anesthetic was discovered in eugenol derivatives such as eunal (acetamidoeugenol or 2-(4-allyl-2-methoxyphenoxy)-*N*,*N*-diethylacetamide), propinal (*N*,*N*-diethyl-2-(2-methoxy-4-propylphenoxy)acetamide) and propanidid (propyl [4-[(*N*,*N*-diethyl-carbamoyl)methoxy]-3-methoxyphenyl]acetate) (Figure 2). Although propanidid was first used as an ultra-short-acting anesthesia-inducing agent for short minor operations, it was withdrawn because of the anaphylactic reaction when given intravenously and orally. James and Glen [12] synthesized a series of alkylphenols including thymol and its structural analogs to determine the structure and anesthetic activity relationship, which indicated that diisopropyl phenol derivatives are promising as an intravenous anesthetic. Among them, propofol (2,6-diisopropylphenol) (Figure 2) was revealed to have desirable clinical features [13]. In 1977, propofol was subjected to clinical trials as a short-acting intravenous agent to induce and maintain general anesthesia, and thereafter it largely replaced thiopental. Propofol is not only structurally related to plant terpenoids but it also allosterically positively modulates GABA_A_ receptors like thymol [14] and eugenol [15]. Propofol, thymol and eugenol also interact mechanistically with lipid membranes to modify the physicochemical properties of biological and biomimetic membranes in a structure-dependent manner [16,17].

## 4. Phytochemicals with the Local Anesthetic Activity

Local anesthetics reversibly block voltage-gated (voltage-dependent or voltage-sensitive) Na^+^ channels (Nav channels) that are responsible for the initiation and propagation of action potentials of excitable cells in the peripheral nervous system and the cardiac system [10]. Voltage-gated Na^+^ channels are integral membrane proteins that are composed of a core α-subunit associated with one or more regulatory β-subunits (β1, β2, β3 and β4). The α-subunit not only forms the pore selectively permeable for Na^+^ ions but also contains the binding or receptor site for local anesthetics, anti-arrhythmic drugs and several neurotoxins. Local anesthetics bind to such a site and cause occlusion of the pore, resulting in the blockade of Na^+^ channels. At least nine distinct α-subunits (Nav1.1 to Nav1.9) have been cloned from mammalian Na^+^ channels. Nav1.7, Nav1.8 and Nav1.9 are the channel isoforms of nociceptive neurons in the peripheral nervous system, and Nav1.1, Nav1.2, Nav1.3 and Nav1.6 are the isoforms in the central nervous system, whereas Nav1.4 and Nav1.5 are in skeletal muscle and heart, respectively [18]. Since Nav1.7 and Nav1.8 isoforms play a crucial role in pain transmission, both channels are implicated as the targets for anesthetic and analgesic drugs. Based on their affinity for neurotoxin tetrodotoxin (TTX), Na^+^ channel subtypes are divided into TTX-sensitive voltage-gated Na^+^ channels (including Nav1.1, Nav1.2, Nav1.3, Nav1.4, Nav1.6 and Nav1.7) and TTX-resistant voltage-gated Na^+^ channels (including Nav1.5, Nav1.8 and Nav1.9), in which Nav1.8 and Nav1.9 are predominantly found in dorsal root ganglion neurons. While specific blockade of single or selected Na^+^ channel subtypes in sensory neurons is considered to induce local anesthesia with less adverse effects, conventional local anesthetics and anti-arrhythmic drugs act on different Na^+^ channel isoforms.

Local anesthetics introduced to clinical practice have a basic structure consisting of a lipophilic aromatic group, a positively chargeable amino terminus and an intermediate chain. In extracellular fluids, their molecules exhibit equilibrium between uncharged and charged forms, which is determined by the p*K*a of drugs and the pH of media. Once local anesthetics diffuse across membrane lipid bilayers, they re-exhibit equilibrium between uncharged and charged forms in intracellular fluids of cytoplasm. Their charged molecules exclusively interact with the receptor sites of Na^+^ channels. In addition to membrane-embedded channel proteins, local anesthetics structure-specifically act on membrane-constituting lipids, directly depressing the functions of neuronal membranes and indirectly inhibiting the activity of Na^+^ channels by modifying the physicochemical properties (fluidity, order, microviscosity or elasticity) of lipid membranes surrounding channel proteins [11]. Since plant terpenoids, alkaloids and flavonoids have amphiphilic structures as well as local anesthetics, these phytochemicals are expected to affect the activity of voltage-gated Na^+^ channels through the common mechanisms.

### 4.1. Plant Preparations

Chewing *Piper betel* (Piperaceae) leaves, called betel in southern India and Malaysia, is known to numb the mouth and dull the taste. Krishnakumar et al., [19] investigated the local anesthetic activity of this plant by an intradermal wheal assay using guinea pigs and a corneal reflex test using rabbits. The plain extracts from betel leaves showed both infiltration and surface anesthetic effects that were almost comparable to those of lidocaine.

The fruit tree *Vitex doniana* (Verbenaceae) growing in an African savanna is used for treating chronic cutaneous wounds. Abdulrahman et al., [20] prepared aqueous extracts from its root bark by a Soxhlet extraction method and examined their effects on the peripheral nervous system of rabbits by an intradermal wheal assay. Injecting extracts of 25 mg/mL and 100 mg/mL produced 55.6% and 80.6% local anesthesia, respectively, although they were less effective than lidocaine of 0.3 mg/mL and 1.0 mg/mL (i.d.). In their following study [21], 95% (*v*/*v*) ethanol extracts from the stem bark of *Vitex doniana* produced 70% and 80% local anesthesia at 50 mg/mL and 100 mg/mL (i.d.), respectively. The ethanol extracts also exhibited the significant antinociceptive activity against acetic acid-induced writhing and thermally-induced pain in mice.

*Spilathes acmella* (Asteraceae), an herb growing throughout the tropics, is used for rheumatism, sore throat and toothache. Eating its leaves and flowers is known to numb the tongue. Chakraborty et al., [22] tested the local anesthetic activity of *Spilathes acmella* by an intracutaneous wheal assay. They injected a 0.2 mL-volume of aqueous extracts obtained from its aerial parts to the backs of guinea pigs and observed the responses of wheal areas to pin prick. Injections of 10% and 20% extracts produced 70% and 87% local anesthesia, respectively, while 2% lidocaine showed 97% local anesthesia.

Karaya gum or sterculia gum from the trunk of *Sterculia tragacantha* (Malvaceae), a deciduous shrub in tropical Africa, is used for treating burns. Udegbunam et al., [23] prepared 80% (*v*/*v*) methanol extracts from *Sterculia tragacantha* leaves to verify the anesthetic activity by an intradermal wheel test using guinea pigs. Injections at 0.03 mg/mL and 10 mg/mL produced 86% and 100% local anesthesia, respectively, compared with 69% and 94% local anesthesia induced by lidocaine of 0.03 mg/mL and 0.1 mg/mL. They fractionated the active extracts by silica-gel chromatography and indicated that alkaloid and saponin components are responsible for local anesthetic effects. They also reported that the subcutaneous injection of *Sterculia tragacantha* methanol extracts induced local anesthesia in goats [24].

### 4.2. Essential Oils and Terpenoids

Essential oils, the concentrated liquids of a highly complex mixture of volatile phytochemicals, are generally extracted from aromatic plants, including citrus peel and caraway. In addition to the utility as perfume and flavor, the medicinal property has been suggested for essential oils from peppermint, lavender, eucalyptus, rosemary, thyme, nutmeg and chamomile. Terpenoids occupy more than 90% of phytochemicals contained in the essential oils. Their structures consisting of five-carbon (C5) isoprene units are classified by the number of C5 unit into monoterpenoids with 2 × C5 unit, sesquiterpenoids with 3 × C5 unit, diterpenoids with 4 × C5 unit and triterpenoids with 6 × C5 unit. Monoterpenoids are composed of acyclic (open-chain), monocyclic and bicyclic structures. Excellent reviews were recently published for the pharmacological effects of essential oils and their component terpenoids by de Sousa [25] and Guimarães et al., [26], and for the therapeutic benefits of essential oils by Djilani and Dicko [27].

**Figure 3:**
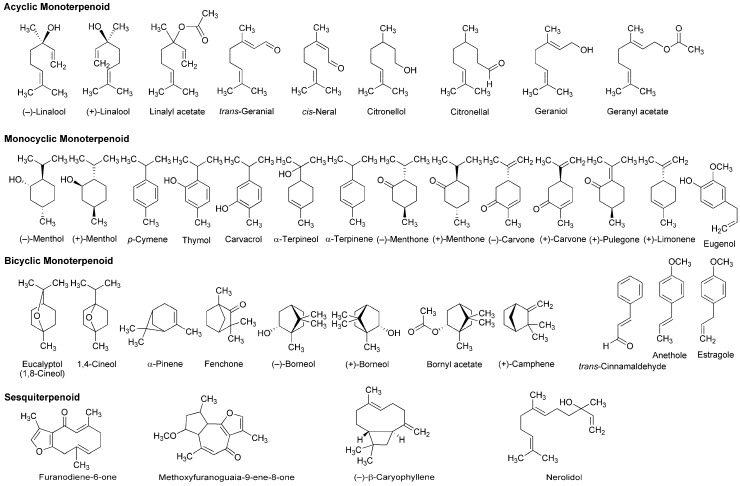
Terpenoids with local anesthetic activity.

A variety of monoterpenoids have been suggested to modulate the activity of voltage- and ligand-gated ion channels [28]. Zalachoras et al., [29] determined the conduction changes of frog sciatic nerve fibers to compare the local anesthetic activity of five monoterpenes: acyclic linalool, monocyclic *p*-cymene, and bicyclic eucalyptol (1,8-cineol), α-pinene and fenchone (Figure 3). They placed the isolated nerves in a three-chambered recording bath and monitored compound action potentials (CAPs). Their results indicated that 7.5–30 mM linalool and 30 mM fenchone exert local anesthetic effects as well as 3.5–30 mM lidocaine, while the effects of eucalyptol, α-pinene and *p*-cymene were minor.

Lavender (*Lavandula angustifolia*, Lamiaceae) has been traditionally used as a medicinal herb with relaxant and antispasmodic activity. Ghelardini et al., [30] showed that lavender essential oil and its major components linalool and linalyl acetate (Figure 3) reduce the electrically evoked contractions of rat phrenic-hemidiaphragm at 0.1 μg/mL to 1 mg/mL as well as procaine and lidocaine. In their following experiments, lavender essential oil, linalool and linalyl acetate depressed rabbit conjunctival reflexes by administrating 0.03–2.5 mg/mL in the conjunctival sac. Such effects were evident 5 min after 2.5 mg/mL administration and diminished within 15 min. Leal-Cardoso et al., [31] revealed that linalool reversely blocks the excitability of rat sciatic nerves and inhibits the voltage-gated Na^+^ currents of rat dorsal root ganglion neurons at sub-micromolar concentrations. Linalool exists as (−)-enantiomer or (+)-enantiomer in essential oils because it has a chiral center at the 3-position (Figure 3). (−)-Linalool predominantly occurs in essential oils from lavender, laurel (*Laurus nobilis*, Lauraceae) and basil (*Ocimum basilicum*, Lamiaceae), but (+)-linalool in ones from coriander (*Coriandrum sativum*, Apiaceae). Peana et al., [32] reported the comparative anti-inflammatory effects of (−)-enantiomer, racemate and acetyl ester of linalool. In a carrageenan-induced hind paw edema model of rats, (−)-linalool (25–75 mg/kg, s.c.) reduced the edemas more effectively than racemic linalool and linalyl acetate.

Monocyclic monoterpene menthol is contained in essential oils from peppermint (*Mentha piperita*, Lamiaceae) and spearmint (*Mentha spicata*, Lamiaceae) that are used for aromatherapy, mouthwash, toothpaste and topical preparations to relieve irritation and inflammation. Among eight stereoisomers, menthol naturally occurs as a (−)-enantiomer of the 1*R*,2*S*,5*R* configuration (Figure 3). Galeotti et al., [33] comparatively evaluated the local anesthetic activity of (−)-menthol and (+)-menthol and their structural analog thymol and (−)-menthone (oxidized menthol). (−)-Menthol and (+)-menthol reduced the electrically evoked contractions of rat phrenic nerve-hemidiaphragm at 0.1–100 ng/mL as well as procaine, although thymol and (−)-menthone were not effective even at 1 μg/mL. When applied to the rabbit conjunctival sac at 30–300 μg/mL, (−)-menthol and (+)-menthol increased the number of stimuli necessary to provoke the reflex, but neither thymol nor (−)-menthone. Both menthol enantiomers produced these effects 5 min after treatments. Gaudioso et al., [34] studied the effects of micromolar menthol on different Na^+^ channel subtypes of rat dorsal root ganglion neurons by a patch clamp method. They indicated that menthol inhibits TTX-resistant Nav1.8 and Nav1.9 and TTX-sensitive Na^+^ channels in a concentration-, voltage- and frequency-dependent manner. Pan et al., [35] examined the action of menthol on pain hypersensitivity induced by inflammation. Their results suggested that menthol is a nonselective analgesic to act on both peripheral and central pain targets.

Kumamoto et al., [36,37,38] carried out a series of studies to compare the effects of various monoterpenoids on nerve conduction and transient receptor potential channels by recording CAPs in frog sciatic nerves. Acyclic citral (a mixture of *trans*-geranial and *cis*-neral), citronellol, citronellal, (−)-linalool, racemic linalool and geraniol; monocyclic (−)-menthol, (+)-menthol, thymol, carvacrol (thymol isomer), α-terpineol, (−)-menthone, (+)-menthone, (−)-carvone, (+)-carvone and (+)-pulegone; terpenoid phenol eugenol; bicyclic (−)-borneol, (+)-borneol, eucalyptol (1,8-cineol) and 1,4-cineol; and terpenoid ester linalyl acetate, geranyl acetate and bornyl acetate (Figure 3) reduced CAP peak amplitudes with IC_50_ values of 0.34–7.2 mM. These monoterpenoids also inhibited nerve conduction by blocking TTX-sensitive voltage-gated Na^+^ channels involved in CAP production, but not activating transient receptor potential channels. The inhibition of CAPs was greatest in carvacrol and thymol, followed by citronellol, bornyl acetate and citral. In the structure and activity relationship, the CAP-inhibitory potency generally varied in the order of being phenols (carvacrol, thymol and eugenol) ≥ aldehydes (citral and citronellal) ≥ esters (bornyl acetate, geranyl acetate and linalyl acetate) ≥ alcohols (citronellol, geraniol, (+)-menthol, (−)-menthol, (+)-borneol, racemic linalool, (−)-borneol, (−)-linalool and α-terpineol) ≥ ketones ((+)-pulegone, (−)-carvone, (−)-menthone, (+)-carvone and (+)-menthone) > bicyclic monoterpenes (eucalyptol and 1,4-cineol). When comparing IC_50_ values (0.34–0.93 mM) to reduce CAP peak amplitudes in frog sciatic nerves, the local anesthetic effects of carvacrol, thymol, citronellol, bornyl acetate, citral, citronellal, geranyl acetate, geraniol, linalyl acetate and (+)-menthol were almost equivalent to those of levobupivacaine, ropivacaine, lidocaine and cocaine [39,40,41].

In in vitro experiments of Joca et al., [42] using rat nerve samples, carvacrol reversely blocked the excitability of sciatic nerves with an IC_50_ value of 0.5 mM and reduced the voltage-gated Na^+^ currents of dorsal root ganglion neurons with an IC_50_ value of 0.37 mM. Cavalcante Melo et al., [43] confirmed its in vivo effects on mice. Carvacrol (50–100 mg/kg, p.o.) significantly inhibited nociception in acetic acid-induced abdominal constriction, formalin injection and hot plate tests. Such effects were not reversed by naloxone and l-arginine, indicating that neither the opioid system nor the nitric oxide pathway is responsible for antinociception by carvacrol. Gonçalves et al., [44] reported that (−)-carvone reduced the excitability of rat sciatic nerves at 10 mM to block about 50% CAP and that (−)-carvone (100–200 mg/kg, i.p.) was effective in inhibiting acetic acid-induced writhing and formalin-induced hind paw nociception of mice.

Leal-Cardoso and his colleagues [45,46,47,48,49,50] performed a series of in vitro experiments using nerve samples from rats to determine the local anesthetic activity of monoterpenoid components in *Croton nepetaefolius* (Euphorbiaceae), *Ocimum basilicum* (Lamiaceae) and *Ravensara anisata* (Lauraceae). These plants used in folk medicine are rich in essential oils containing estragole and anethole (Figure 3). Both monocyclic monoterpenes directly inhibited Na^+^ channels and blocked the excitability of peripheral nerves. Estragole inhibited total Na^+^ currents and TTX-resistant Na^+^ currents in dorsal root ganglion neurons with IC_50_ values of 3.2 and 3.6 mM, respectively, suggesting that it affects the excitability of peripheral nerves as well as local anesthetics [46]. Among monoterpenoids contained in *Lippia alba* (Verbenaceae) and *Croton nepetaefolius* (Euphorbiaceae), citral inhibited CAPs in sciatic nerves with an IC_50_ value of 0.23 mM [48]. Eucalyptol reversely blocked the excitability of sciatic nerves and superior cervical ganglion neurons by acting on Na^+^ channels directly [49,50].

Ghelardini et al., [51] compared the local anesthetic activity of components in essential oils from medicinal herb marjoram (*Origanum majorana*, Lamiaceae) and anise (*Pimpinella anisum*, Apiaceae) by in vitro and in vivo experiments. α-Terpineol and anethole reduced the electrically evoked contractions of rat phrenic nerve-hemidiaphragm at 0.001–1 μg/mL in a concentration-dependent manner, but not citronellal, (−)-carvone, (+)-carvone, α-terpinene, eugenol and *trans*-cinnamaldehyde (Figure 3). Both active monoterpenes also increased the number of stimuli required to evoke rabbit conjunctival reflex at 10–100 μg/mL. Quintans-Júnior et al., [52] examined in vivo effects of several monoterpenes by acetic acid-induced writhing and formalin-injected hind paw licking tests using mice. *para*-Cymene (50–200 mg/kg, i.p.) showed the greatest antinociceptive effect in both tests, followed by acyclic geranyl acetate and bicyclic (+)-camphene (Figure 3).

Myrrh, a resin secreted by plants of the genus *Commiphora* (Burseraceae), has traditionally been used for treating wounds and toothache. Dolara et al., [53] chromatographed the extracts from *Commiphora molmol* to specify one fraction (280 μg/mL) with about a half anesthetic potency of 1% procaine by a rabbit conjunctival reflex test. They isolated two phytochemicals from the active fraction and identified them as furanodiene-6-one and methoxyfuranoguaia-9-ene-8-one (Figure 3). Both sesquiterpenes selectively and reversibly blocked Na^+^ channels in an electrophysiological experiment using rat cardiac myocytes.

The bicyclic sesquiterpene (−)-β-caryophyllene (Figure 3) naturally occurs in essential oils from clove (*Syzygium aromaticum*, Myrtaceae), hop (*Humulus lupulus*, Cannabaceae), wild sweet basil (*Ocimum campechianum*, Lamiaceae) and oregano (*Origanum vulgare*, Lamiaceae). Ghelardini et al., [54] reported that β-caryophyllene exerted in vitro and in vivo local anesthetic effects comparable to those of procaine. Beta-caryophyllene reduced the electrically evoked contractions of rat phrenic nerve-hemidiaphragm at 0.1 ng/mL to 1.0 μg/mL and increased the number of stimuli necessary to provoke rabbit conjunctival reflex at 10 μg/mL to 1.0 mg/mL.

As one of pharmacological mechanisms for terpenoids, Mendanha et al., [55] investigated the interaction with biological membranes by electron paramagnetic resonance spectroscopy. All the tested nerolidol, menthol, pulegone, carvone, (+)-limonene, α-terpineol and eucalyptol (Figure 3) mechanistically interacted with mouse fibroblast and human erythrocyte membranes to increase their fluidity with the potency being sesquiterpene nerolidol greater than other monoterpenoids. Yin et al., [56] and Nowotarska et al., [57] revealed that bicyclic borneol and monocyclic carvacrol act on phospholipid bilayers to cause membrane fluidization as well as geraniol. Reiner et al., [17] verified the interactivity of thymol, eugenol and carvacrol with egg phosphatidylcholine unilamellar vesicles by ^1^H-nuclear magnetic resonance spectroscopy. Their results indicated that these monoterpenoids are inserted into lipid bilayers to locate in the region between the choline polar group, the glycerol and the first atoms of the acyl chains. Tsuchiya and Mizogami [58] characterized the membrane effects of terpenoid phenols that increase the fluidity of neuro-mimetic membranes at 1–10 μM with the relative potency being thymol > carvacrol > eugenol as well as 50–200 μM bupivacaine and lidocaine. These phytochemicals penetrated into lipid bilayers with preference to the deeper hydrophobic region of membranes. Thymol and eugenol achieve the concentrations of 10–100 μM in dental pulps and dentins adjacent to the pulp space. Since both terpenoids show local anesthetic and analgesic effects at such micromolar concentrations together with acting on neuronal membranes, they are frequently applied to clinical dentistry as a sedative for toothache, pulpitis and dental hyperalgesia.

Sarmento-Neto et al., [59] recently published a review that focused on the antinociceptive potentials of essential oils from 31 plant species and their major component terpenoids.

### 4.3. Alkaloids

Unlike conventional local anesthetics to act on Na^+^, K^+^ and Ca^2+^ channels, neurotoxins specifically block voltage-gated Na^+^ channels [60], so they should be an ideal local anesthetic. Natural neurotoxins, many of which belong to plant alkaloids, potentially produce long-duration local anesthesia. Some alkaloids isolated from plants of the genera *Aconitum* and *Delphinium* have been applied as analgesic and anti-inflammatory agents in Chinese medicine.

**Figure 4:**
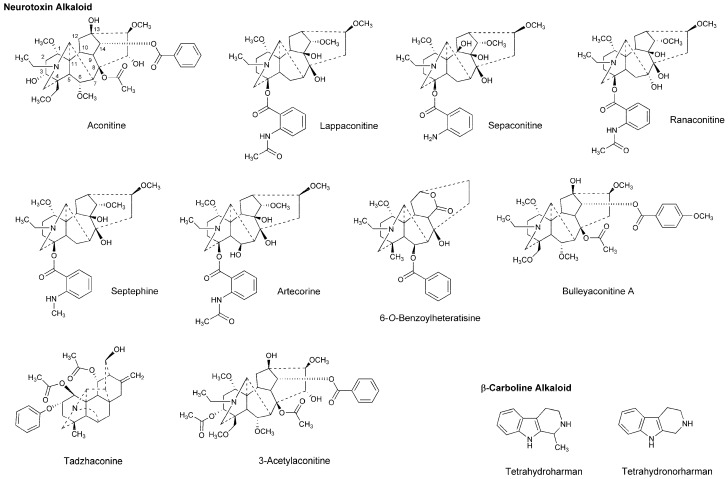
Alkaloids with local anesthetic activity.

Dzhakhangirov et al., [61] investigated the surface and infiltration anesthetic effects of *Aconitum* and *Delphinium* alkaloids by a rabbit corneal reflex test to drop sample solutions (0.01–1%) into the conjunctival sac and by a cat neck trunk anesthesia method to inject 0.1 mL sample solutions (0.1–0.5%) intracutaneously and subcutaneously. Lappaconitine, sepaconitine, ranaconitine, septephine, artecorine, 6-*O*-benzoylheteratisine and tadzhaconine (Figure 4) showed greater potency and longer duration of anesthesia than procaine and lidocaine.

At least six receptor sites on voltage-gated Na^+^ channels have been identified for neurotoxin alkaloids. Plant alkaloid aconitine, lappaconitine and bulleyaconitine A (Figure 4) bind to the receptor site 2 to block Na^+^ conduction, whereas pufferfish toxin TTX and shellfish toxin saxitoxin, to the receptor site 1. Site 2 neurotoxins and local anesthetics have overlapping but non-identical binding regions [62]. The most abundant neurotoxins in *Aconitum* and *Delphinium* plants are aconitine-like alkaloids that activate Na^+^ channels and shift a conformational equilibrium toward the activated state, whereas the other alkaloids block voltage-gated Na^+^ channels. Despite the structural similarity, aconitine activates voltage-gated Na^+^ channels, but lappaconitine blocks. The structure and activity relationship indicates that alkaloids with a benzoyl ester side chain at the 14-position and at the 4-position act as an agonist and a blocker of voltage-gated Na^+^ channels, respectively.

Gutser et al., [63] compared the local anesthetic effects of *Aconitum* alkaloids. They intravenously administered alkaloid samples to mice and after 5 min, injected 5% formaldehyde (20 μL, s.c.) to induce hyperalgesia, followed by monitoring nociception-related behaviors during the subsequent 30 min. Antinociceptive ED_50_ values in the early phase (0–15 min) and the late phase (15–30 min) were 0.028 mg/kg and 0.027 mg/kg for aconitine and 0.097 mg/kg and 0.077 mg/kg for 3-acetylaconitine. Both alkaloids showed high affinity for the site 2 of rat synaptosomal Na^+^ channels. In contrast, lappaconitine was less effective to show antinociceptive ED_50_ values of 2.7 mg/kg and 2.9 mg/kg in the early phase and the late phase, respectively. Aconitine and 3-acetylaconitine are speculated to inhibit neuronal conduction by persistent depolarization, whereas lappaconitine, to block Na^+^ channels like local anesthetics. Wang et al., [64] reported analgesic and anti-inflammatory effects of lappaconitine, which were verified by a mouse acetic acid-induced writhing test, a mouse hot plate test, and rat paw and mouse ear edema models.

Wang et al., [65] confirmed the in vitro effects of bulleyaconitine A on neuronal voltage-gated Na^+^ channels under the whole cell patch-clamp configuration of rat pituitary GH_3_ cells expressing Nav1.1, 1.2, 1.3 and 1.6 isoforms. Next, they injected bulleyaconitine A in a volume of 0.2 mL into the sciatic notch of the left hind limb of rats after inhalational anesthesia with sevoflurane and determined changes in sensory and motor functions. Bulleyaconitine A blocked both sensory and motor function of the sciatic nerves at 0.375 mM, although it induced hyperexcitability and cardiac arrhythmia following the sciatic nerve block. Co-injection of 0.375 mM bulleyaconitine A with 2% lidocaine or epinephrine (1:100,000) decreased such systemic toxicity and prolonged the nerve-blocking duration to ~4 h. In their following study [66], the effects of bulleyaconitine A on voltage-gated Na^+^ channels were evaluated by recording Na^+^ currents of human embryonic kidney cells expressing different Nav isoforms and by measuring the cutaneous trunci muscle reflex after subcutaneous (via dorsal skin) injection of 0.6 mL test solution to rats. Bulleyaconitine A blocked Nav1.7 and Nav1.8 Na^+^ currents at 10 μM and induced the complete nociceptive blockade lasting for ~3 h at 0.125 mM. When co-injecting 0.125 mM bulleyaconitine A with 0.5% lidocaine/epinephrine (1:200,000), the duration of cutaneous analgesia was increased from 3 h to 24 h without adverse effects.

With respect to local anesthetic *Aconitum* and *Delphinium* alkaloids, Turabekova et al., [67] recently published an excellent report on the relationship between structure and activity to antagonize voltage-gated Na^+^ channels.

Tsuchiya [68] examined the membrane effects of β-carboline alkaloids on lipid bilayer membranes by a fluorescence polarization method. He demonstrated that tetrahydroharman and tetrahydronorharman (Figure 4) interact with biomimetic membranes to show biphasic effects that increase the membrane fluidity at high micromolar concentrations but decrease at low nanomolar concentrations. There is the possibility that these membrane-interacting alkaloids may counteract the mechanistic membrane effects of local anesthetics to affect their anesthetic efficacy at physiologically-relevant ~15 nM [69].

### 4.4. Flavonoids

**Figure 5:**
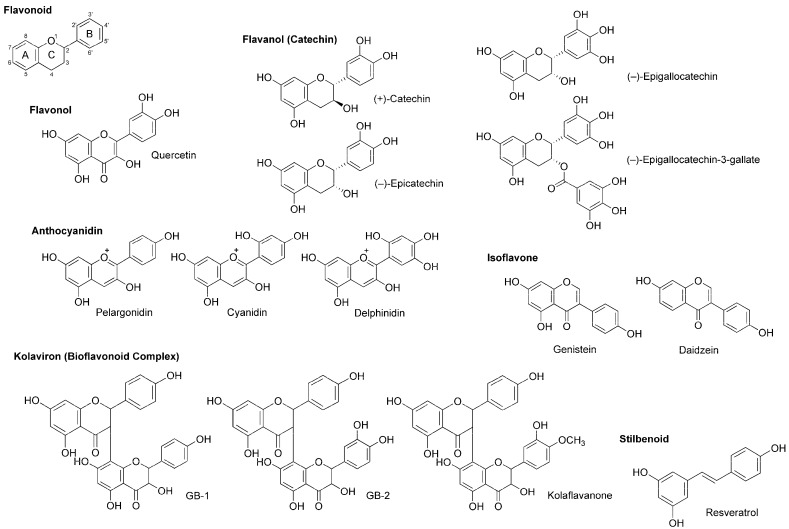
Polyphenolic flavonoids and stilbenoid with local anesthetic activity.

Flavonoids are polyphenolic phytochemicals that ubiquitously occur in edible and medicinal plants. A number of flavonoids are structurally derived from the parent compound with a tricyclic (C_6_–C_3_–C_6_) skeleton, sharing a benzene ring A condensed with a heterocyclic six-membered ring C that carries a phenyl ring B at the 2-position for flavonoids (Figure 5). Based on a structural variation of C ring and its substituents, flavonoids are divided into several subclasses of flavone, flavonol (3-hydroxyflavone), flavanone (dihydroflavone), flavanonol (dihydroflavonol), flavanol (catechin), anthocyanidin with the backbone of 1-benzopyrylium instead of 1-benzopyran-4-one, chalcone lacking the C ring, and isoflavone with the B ring at the 3-position.

Wu et al., [70] recorded patch-clamp whole-cell currents of mouse dorsal root ganglionic neuronal cells and demonstrated that polyphenols extracted from red wine inhibit voltage-gated Na^+^, K^+^ and Ca^2+^ channels with IC_50_ values of 2.5, 4.0 and 0.8–1.5 μg/mL, respectively. They referred to active substances as flavonoids like quercetin, (+)-catechin, (−)-epicatechin and anthocyanins (mostly 3-glucosides of anthocyanidins) and the stilbenoid resveratrol (Figure 5).

(−)-Epigallocatechin-3-gallate and (−)-epigallocatechin (Figure 5) are best known as the active components in green tea (the product of *Camellia sinensis*, Theaceae), which possess antiproliferative, antitumor, antimicrobial, antithrombotic, anti-inflammatory, anti-allergic, apoptosis-inducing and antioxidant property. Kim et al., [71] examined their effects on Na^+^ currents in rat dorsal root ganglion neurons. (−)-Epigallocatechin-3-gallate inhibited both TTX-sensitive and TTX-resistant Na^+^ currents more potently than (−)-epigallocatechin.

In in vitro studies of Paillart et al., [72], genistein and daidzein (Figure 5) blocked voltage-sensitive Na^+^ channels in cultured rat brain neurons with IC_50_ values of 60 and 195 μM, respectively. Their effects were not mediated by tyrosine kinase inhibition that is well-known as the pharmacological mechanism of isoflavonoids.

*Garcinia kola* (Guttiferae) is a medicinal plant used for laryngitis, cough and liver disease. Its seeds predominantly contain kolaviron [73], a bioflavonoid complex consisting of 3,8-linked flavanone dimers such as GB-1, GB-2 and kolaflavanone (Figure 5). Tchimene et al., [74] evaluated the local anesthetic activity of ethanol extracts from *Garcinia kola* seeds and their flavonoid components by an intradermal wheal assay using guinea pigs. GB-1 induced 92% local anesthesia at 10 mg/mL (i.d.), being comparable to the effect of lidocaine (0.66 mg/kg, i.d.).

### 4.5. Stilbenoids

Besides flavonoids, stilbenoids are also part of natural polyphenols contained in plants. The best known polyphenolic stilbene is resveratrol (Figure 5). Resveratrol is referred to as a phytoalexin that plants biosynthesize by pathogen infection and accumulate at the infected area to exhibit the antimicrobial activity for protection. Japanese knotweed (*Fallopia japonica*, Polygonaceae), grapevines and berry fruits such as blueberry, cranberry and bilberry are rich in resveratrol. Since these plants have anti-inflammatory, neuroprotective and cancer preventive property, Kim et al., [75] investigated the effects of resveratrol on TTX-sensitive and TTX-resistant Na^+^ channels implicated in pain pathways. Resveratrol suppressed both types of Na^+^ currents in rat dorsal root ganglion neurons with preference for TTX-sensitive Na^+^ channels.

## 5. Phytochemicals with the General Anesthetic Activity

Although the exact pharmacological mechanisms of general anesthesia are still an open question, currently used general anesthetics and anesthesia-related drugs (sedatives, anxiolytics or anesthetic adjuncts) are considered to target inhibitory GABA_A_ receptors and excitatory NMDA receptors [76].

Inhibitory neurotransmitter GABA binds to GABA_A_ receptors that are ligand-gated Cl^−^ channels to allow the influx of Cl^−^ into postsynaptic neurons, inhibiting the neuronal excitability through synaptic phase currents and extrasynaptic tonic currents [77]. GABA_A_ receptors are expressed in various neurons of cortex, hippocampus, cerebellum and olfactory bulb. While heteromeric GABA_A_ receptors are composed of α and β subunits, there are distinct subunit combinations in which α1β2γ2 (the most abundant receptor isoform) and α2β3γ2 are primarily responsible for sedative and anxiolytic effect, respectively, or for both. Intravenous agents such as propofol, etomidate, benzodiazepines and barbiturates, and inhalational agents such as halothane, isoflurane, sevoflurane and nitrous oxide act as a positive allosteric modulator and a direct activator of GABA_A_ receptors at clinically relevant concentrations to produce general anesthesia, sedation, anxiolysis and cessation of convulsions. GABA_A_ receptors have specific binding sites for GABA and many drugs including benzodiazepines, barbiturates, volatile anesthetics, picrotoxinin, neuro-active steroids and ethanol. The GABAergic system is also mechanistically associated with the effects of volatile odorants contained in essential oils.

NMDA receptors consisting of an NR1 subunit combined with NR2 subunits are activated by glutamate and glycine to open the ion channels nonselective to positively charged cations, resulting in neuronal excitation. Ketamine and nitrous oxide act on NMDA receptors as a non-competitive antagonist and a channel blocker, respectively, to show analgesic, sedative and anesthesia-maintaining effects. Activation of NMDA subtypes of glutamate receptors is also associated with cell damages caused by neuronal ischemia. Therefore, antagonism or inhibition of NMDA receptors is considered to lead to not only dissociative anesthesia but also possible neuroprotection.

In addition to receptor proteins, general anesthetics act on membrane-constituting lipids to modify organization, dynamics and physicochemical properties of biomembranes, resulting in direct disturbance of the function of neuronal membranes and indirect modulation of the receptor activity through a conformational change of membrane-embedded proteins [78]. Propofol preferentially locates in the hydrophobic deeper regions of membrane lipid bilayers to change the fluidity, order, permeability and dynamics of neuronal membranes with the potency correlating to GABA_A_ receptor-modulatory effects. Such a membrane interaction mechanism, at least partly but significantly, underlies the effects of intravenous anesthetics, barbiturates, benzodiazepines and ketamine.

Among phytochemicals, terpenoids, flavonoids and alkaloids are expected as the neuro-active lead compounds for general anesthetics and anesthesia-related drugs because they are able to interact with GABA_A_ receptors, NMDA receptors and lipid membranes [78,79].

### 5.1. Plant Preparations

Besides exhibiting the local anesthetic and antinociceptive activity, black plum (*Vitex doniana*, Verbenaceae) has been speculated to have some effect on the central nervous system. In rat in vivo experiments of Abdulrahman et al., [20] and Tijjani et al., [21], aqueous extracts (50–400 mg/kg, i.p.) from the root bark and ethanol extracts (100–200 mg/kg, i.p.) from the stem bark potentiated the effects of thiopental (20 mg/kg, i.p.) and pentobarbital (35 mg/kg, i.p.), respectively.

Zaku et al., [80] reported that aqueous extracts (100–200 mg/kg, i.p.) from the root bark of medicinal plant *Ficus sycomorus* (Moraceae) increased the effect of aminobarbitone (12 mg/kg, i.p.) on rats. They specified phytochemicals present in the active extracts as terpenoids, alkaloids and flavonoids.

Plants of the genus *Passiflora* consist of many species, including edible and medicinal plants such as *Passiflora incarnata*, *Passiflora quadrangularis* and *Passiflora actinia* that are used worldwide for anxiety, neuralgia and insomnia. The most popular species, *Passiflora incarnata* (Passifloraceae), is known as purple passionflower and maypop with the analgesic, anxiolytic, sedative and anticonvulsant activity. Grundmann et al., [81] neuropharmacologically studied the effect of commercially available 50% ethanol extract from *Passiflora incarnata* by an elevated plus maze test using mice. The extract (375 mg/kg, p.o.) exerted an anxiolytic effect comparable to that of diazepam (1.5 mg/kg, p.o.). This effect was inhibited by GABA_A_-benzodiazepine receptor antagonist flumazenil (3 mg/kg, i.p.), suggesting the mechanistic contribution of the GABAergic system. In in vitro experiments of Appel et al., [82], the same *Passiflora incarnata* extract inhibited GABA uptake into rat cortical synaptosomes with an EC_50_ value of 95.7 μg/mL and competed with a specific antagonist for binding to GABA_A_ receptors of rat brain membranes with an IC_50_ value of 101 μg/mL. Lolli et al., [83] used another species *Passiflora actinia* (Passifloraceae) to determine the GABA_A_ receptor-modulatory activity. Methanol and hydroalcoholic extracts from its leaves showed anxiolytic-like effects at 100–600 mg/kg (p.o.) in an elevated plus maze test using mice, which were blocked by GABA_A_-benzodiazepine receptor antagonist flumazenil (10 mg/kg, i.p.).

### 5.2. Essential Oils and Terpenoids

Etomidate, barbiturates, benzocaine and lidocaine are used to immobilize aquatic animals and reduce their stress and mortality. In recent years, attention has been paid to the anesthetic effects of plant products because they have the relatively low toxicity compared with conventional synthetic drugs. A series of experiments of Heinzmann et al., in which fishes and crustaceans were submitted to aquaria containing essential oils, revealed that silver catfish and white shrimp are anesthetized by essential oils from *Lippia alba* (Verbenaceae) [84], *Aloysia triphylla* (Verbenaceae) [85], *Hyptis mutabilis* (Lamiaceae) [86] and *Hesperozygis ringens* (Lamiaceae) [87]. The GABAergic system was suggested to underlie their anesthetic effects. However, whether these results are applicable to mammals and humans is unknown.

**Figure 6:**
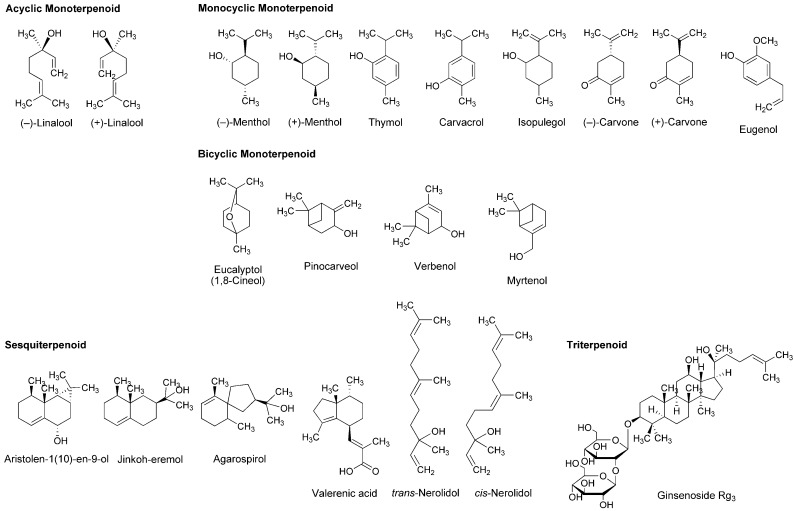
Terpenoids with general anesthetic activity.

Aromatherapy is traditionally utilized for managing pain, anxiety, depression and stress because the aromas of essential oils are very likely to influence emotional responses. Agarwood (“Jinkoh” in Japanese) is a resinous portion of *Aquilaria* trees in the family Thymelaeaceae and spikenard (“Kanshokoh” in Japanese) is dried root and rhizome of *Nardostachys chinensis* (Valerianaceae). Both plant preparations are used not only for oriental incense and scented sachet but also as an herbal tranquilizer. Takemoto et al., [88] examined the neuro-activity of agarwood oils and spikenard extracts by a spontaneous vapor administration system and found that mice were sedated by inhaling their vapors. Volatile compounds isolated from the active vapors were also effective at lower concentrations. They identified 10 sesquiterpenes contained in the essential oil prepared from spikenard. When comparing these sesquiterpenes, aristolen-1(10)-en-9-ol (Figure 6) most potently inhibited the locomotion of caffeine-treated mice by approximately 60% at 300 μg/cage [89]. Inhalation of aristolen-1(10)-en-9-ol prolonged pentobarbital (30 mg/kg, i.p.)-induced sleeping time of mice with the potency comparable to that of diazepam (1 mg/kg, p.o.). This effect was inhibited by GABA_A_-benzodiazepine receptor antagonist flumazenil (3 mg/kg, i.p.), suggesting that the GABAergic system is associated with the neuro-activity of aristolen-1(10)-en-9-ol. Okugawa et al., [90] also reported that agarwood sesquiterpene jinkoh-eremol and agarospirol (Figure 6) showed neuroleptic effects on mice. De Sousa et al., [91] recently published a systematic review about the anxiolytic-like effects of essential oils from *Lavendula angustifolia* (Lamiaceae), *Citrus aurantium* (Rutaceae), *Alpinia zerumbet* (Zingiberaceae) and other aromatic plants.

Plants of the genus *Sideritis* (Lamiaceae) are used to make tea with the sedative and antioxidant property. Kessler et al., [92] identified different terpenoids in the extracts from *Sideritis arguta*, *Sideritis condensata*, *Sideritis stricta* and *Sideritis sipylea*. They investigated the effects of these *Sideritis* terpenoids on GABA_A_ receptor α1β2 or α1β2γ2 subunits expressed in *Xenopus laevis* oocytes or human embryonic kidney cells. Monocyclic isopulegol and bicyclic pinocarveol, verbenol and myrtenol (Figure 6) significantly modulated GABA_A_ receptor functions at 3–300 μM independently of the γ2 subunit as well as general anesthetics.

The root extract of valerian, a perennial flowering plant of the genus *Valerian*, is utilized as a sedative or tranquilizer in herbal medicine. *Valeriana officinalis* (Valerianaceae) most widely used in Europe and USA contains sesquiterpene valerenic acid (Figure 6). Yuan et al., [93] demonstrated that a commercially available valerian extract and valerenic acid inhibit GABA_A_ receptor agonist muscimol-sensitive neurons in neonatal rat brainstem preparations with IC_50_ values of 240 μg/mL and 23 μM, respectively. These effects were antagonized by GABA_A_ receptor antagonist bicuculline at 10 μM. Khom et al., [94] and Traune et al., [95] reported that valerenic acid allosterically subunit-specifically modulated GABA_A_ receptors and enhanced Cl^−^ currents through GABA_A_ receptors expressed in *Xenopus laevis* oocytes. By a radioligand binding assay with crude rat brain membranes, Benke et al., [96] revealed that valerenic acid allosterically interacts with benzodiazepine and GABA binding sites of GABA_A_ receptors. In the in vivo experiments, valerenic acid (1–6 mg/kg, i.p. and 10 mg/kg, p.o.) also showed anxiolytic effects on mice.

In addition to local anesthetic and anti-inflammatory effects [32], acyclic monoterpene (−)-linalool (Figure 6) exhibits the antinociceptive activity associated with the blockade of NMDA receptors and the activation of cholinergic, opioidergic and dopaminergic systems [97], but not with the modulation of GABA_A_ receptors [98]. Linck et al., [99] placed mice for 60 min in a chamber of an atmosphere saturated with 1% or 3% racemic linalool. Inhaling linalool increased the time of sleep induced by intraperitoneal pentobarbital administration. Sugawara et al., [100] compared lavender oil (−)-linalool, coriander oil (+)-linalool and racemic linalool, and concluded that (−)-linalool is most effective in sedating humans. Heldwein et al., [101] indicated that (+)-linalool from *Lippia alba* (Verbenaceae) is primarily responsible for sedation and anesthesia of silver fish.

Watt et al., [102] examined the effect of monocyclic monoterpene menthol on recombinant human GABA_A_ receptors expressed in *Xenopus laevis* oocytes. Menthol enhanced sub-maximal GABA currents at 3–300 μM and shared the site of action on GABA_A_ receptors with propofol. Lau et al., [103] investigated the effect of menthol on GABA_A_ receptor-mediated currents of rat midbrain periaqueductal grey neurons by a whole-cell voltage clamp recording experiment. They showed that menthol prolongs at 150–750 μM the duration of spontaneous inhibitory postsynaptic potentials mediated by GABA_A_ receptors.

Reiner et al., [15] studied the action of terpenoid phenols on primary cultures of mouse cortical neurons. Thymol, carvacrol and eugenol enhanced the binding of [^3^H]flunitrazepam to GABA_A_ receptors with micromolar EC_50_ values and increased 2 μM GABA-evoked Cl^−^ influx at micromolar concentrations. Priestley et al., [104] showed that thymol potentiates EC_20_ GABA responses at 1–100 μM by using human GABA_A_ receptor subunit combinations expressed in *Xenopus laevis* oocytes. García et al., [14] evaluated the positive allosteric modulatory effect of thymol on GABA_A_ receptors in primary cultures of mouse cortical neurons. Thymol enhanced 5 μM GABA-induced Cl^−^ influx with an EC_50_ value of 12 μM and directly enhanced the Cl^−^ influx with an EC_50_ value of 135 μM. Its direct effect was inhibited by competitive and non-competitive GABA_A_ receptor antagonists. While phenol derivatives with aliphatic substituents at the *ortho*-position commonly have the property to activate GABA_A_ receptors directly, propofol (2,6-diisopropylphenol) most potently activated Cl^−^ currents via rat GABA_A_ receptors heterologously expressed in human embryonic kidney cells with an EC_50_ value of 23 μM, followed by thymol (2-isopropyl-5-methylphenol) with an EC_50_ value of 200 μM [105].

The triterpenoid ginsenosides Rb_1_, Rb_2_, Rc, Re, Rf, Rg_1_, Rg_2_, Rg_3_, Rh_1_ and Rh_2_ are contained in ginseng that is traditionally used for improving mood, vitality and sexual function and for treating depression, anxiety and memory problem. Kim et al., [106] revealed that total ginsenosides in ginseng inhibit 100 μM NMDA-produced Ca^2+^ increase in cultured rat hippocampal neurons with an IC_50_ value of 26.6 μg/mL. When comparing several ginsenosides, ginsenoside Rg_3_ (Figure 6) was most effective in attenuating the NMDA receptor-mediated Ca^2+^ influx to show an IC_50_ value of 3.8 μM. Such effects may be related to neuroprotection because injuries of the central nervous system trigger an over release of glutamate that causes the excessive activation of glutamate receptors of NMDA subtypes.

Membrane lipids regulate GABA_A_ receptors, NMDA receptors and ion channels embedded in biomembranes [107]. The pharmacological effects of anesthetics and anesthesia-related drugs are induced by not only the interactions with receptor and channel proteins to modulate their activity but also the interactions with membrane-constituting lipids to alter lipid environments surrounding these functional proteins. Reiner et al., [17] demonstrated that monoterpene thymol, eugenol and carvacrol act on phospholipid multilamellar vesicles to increase the membrane fluidity at 50–200 µM together with modulating GABA_A_ receptors positively as well as intravenous anesthetic propofol. Tsuchiya and Mizogami [108] compared membrane effects of thymol, eugenol and their structurally-related anesthetics including propofol. Propofol most potently interacted with neuro-mimetic membranes at 0.1–10 µM, followed by thymol and eugenol. Mendanha et al., [55] reported that sesquiterpene nerolidol interacted with erythrocyte and fibroblast membranes to change their fluidity more significantly than racemic menthol, racemic carvone and eucalyptol (Figure 6). While ginkgo contains diterpenoids and sesquiterpenoids, in vivo experiments of DeFeudis and Drieu [109] showed that oral administration to mice of the extracts from *Ginkgo biloba* leaves (100 mg/kg/day) for three weeks increases the fluidity of neuronal membranes simultaneously with improving the short-term memory in a passive avoidance paradigm. The membrane interactions of terpenoids are associated with depression of the central nervous system and potentiation of general anesthetic effects [15].

### 5.3. Alkaloids

**Figure 7:**
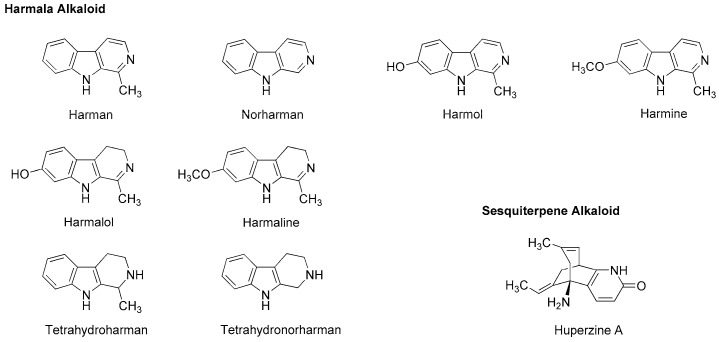
Alkaloids with general anesthetic activity.

Passionflower is the general term for plants of the genus *Passiflora*, of which *Passiflora incarnata* (Passifloraceae) is the most popular species, followed by *Passiflora edulis* (Passifloraceae) and *Passiflora foetida* (Passifloraceae). Its leaves, roots and tea products are used for insomnia, anxiety, neuralgia, epilepsy, hysteria, ulcers, burns and inflammation. Alkaloid components are presumed to be responsible for these effects through GABA_A_ receptor modulation. They are very likely to belong to harmala alkaloids that were originally found in *Peganum harmala* (Nitrariaceae) and identified as phytochemicals with the common β-carboline structure like harman, harmine, harmaline, harmol and halmalol (Figure 7). When Aricioglu and Altunbas [110] administered harman (2.5–10 mg/kg, i.p.) to rats, the time of immobility in a forced swim test and the time spent in open arms in an elevated plus maze were decreased and increased, respectively. Farzin and Mansouri [111] also reported that harman (5–15 mg/kg, i.p.), norharman (2.5–10 mg/kg, i.p.) and harmine (5–15 mg/kg, i.p.) decreased the time of immobility of mice. These effects were inhibited by GABA_A_-benzodiazepine receptor antagonist flumazenil (5 mg/kg, i.p.).

Neuro-active harmala alkaloids such as tetrahydroharman and tetrahydronorharman (Figure 7) also have the property to act on membrane lipids. Tsuchiya [68] investigated their effects on biomimetic phospholipid bilayer membranes to verify one of possible pharmacological mechanisms. Tetrahydroharman showed a concentration-dependent biphasic effect to increase the membrane fluidity at ≥100 μM but decrease the membrane fluidity at ≤1.0 μM. Since tetrahydroharman inhibits the mechanistic membrane effect of propofol at nanomolar concentrations, this alkaloid may potentially affect the anesthetic efficacy of propofol [112].

Chinese herb *Huperzia serrata* (Huperziaceae) contains sesquiterpene alkaloid huperzine A (Figure 7). By whole-cell voltage clamp recording in CA1 pyramidal neurons dissociated from rat hippocampus, Zhang and Hu [113] revealed that huperzine A non-competitively inhibits NMDA-induced currents with an IC_50_ value of 126 µM in a voltage- and use-independent manner. This alkaloid may be effective for neurodegenerative diseases as a non-competitive antagonist of NMDA receptors.

### 5.4. Flavonoids

**Figure 8:**
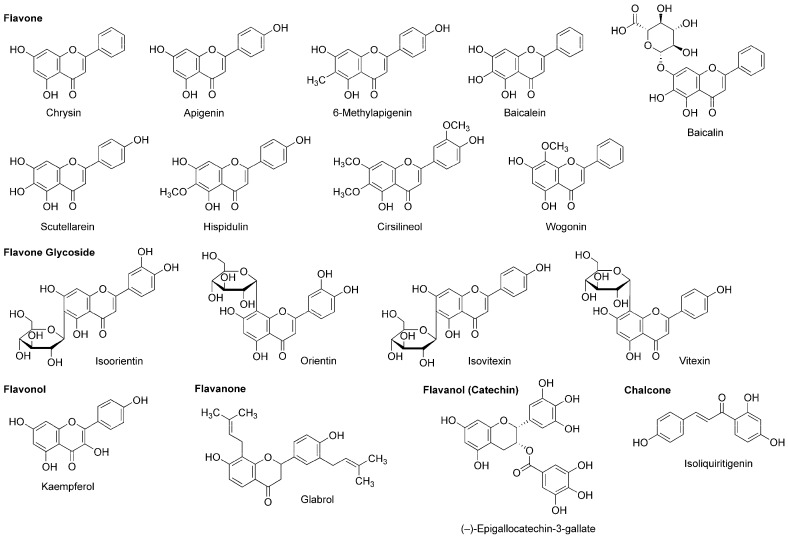
Flavonoids with general anesthetic activity.

A variety of flavonoids have been suggested to act as an allosteric modulator of GABA_A_ receptors [114]. Grundmann et al., [81] reported that the anxiolytic effect of *Passiflora incarnata* extracts was inhibited by GABA_A_-benzodiazepine receptor antagonist flumazenil. They identified the isolated active components as flavone glycoside isoorietin (luteolin-6-*C*-glucoside), orietin (luteolin-8-*C*-glucoside), isovitexin (apigenin-6-*C*-glucoside) and vitexin (apigenin-8-*C*-glucoside) (Figure 8).

While chamomile is the common name for several plant species of the family Asteraceae, tea made from *Matricaria recutita* (Asteraceae) is used for treating anxiety, stress, insomnia and inflammation. Apigenin (Figure 8) contained in such tea has the property of a benzodiazepine partial agonist. Jäger et al., [115] fractionated the extract from *Tanacetum parthenium* (Asteraceae) by flumazenil binding assay-guided chromatography and isolated apigenin with the affinity for a GABA_A_-benzodiazepine site. Wasowski et al., [116] isolated another sedative flavone 6-methylapigenin (Figure 8) from *Valeriana wallichii* (Valerianaceae) by flunitrazepam competitive assay-guided chromatography. 6-Methylapigenin showed higher affinity for GABA_A_ receptors than apigenin.

(−)-Epigallocatechin-3-gallate (Figure 8) is the most active flavanol contained in green tea, the product of *Camellia sinensis* (Theaceae). Park et al., [117] suggested that its anxiolytic and sedative effects are mediated by GABA_A_ receptors. In their in vitro and in vivo experiments, (−)-epigallocatechin-3-gallate (5–20 mg/kg, p.o.) prolonged the sleeping time of mice induced by pentobarbital (42 mg/kg, i.p.) together with increasing the Cl^−^ influx in primary cultured cerebellar cells. Campbell et al., [118] compared the effects of flavonoids on recombinant human GABA_A_ receptors expressed in *Xenopus laevis* oocytes and demonstrated that 0.1 µM (−)-epigallocatechin-3-gallate and 8 µM apigenin enhance 5 µM GABA-induced receptor activation and 3 µM diazepam-induced positive receptor modulation by up to 52% and 22%, respectively.

Salah and Jäger [119] isolated hispidulin and cirsilineol (Figure 8) from a perennial shrub *Artemisia herba-alba* (Asteraceae). In their in vitro study, these flavones acted on GABA_A_-benzodiazepine receptors with IC_50_ values of 8 µM for hispidulin and 100 µM for cirsilineol.

In addition to neuro-active β-carboline alkaloids, plants of the genus *Passiflora* contain sedative flavonoids such as apigenin, chrysin and kaempferol (Figure 8). Because aqueous extracts (100–300 mg/kg, p.o.) from the pericarp of *Passiflora quadrangularis* (Passifloraceae) prolonged the sleep duration of mice in an ethyl ether-induced hypnosis test, Gazola et al., [120] evaluated the neurological effects of their main component apigenin. Apigenin (0.6 mg/kg, p.o.) sedated mice and this effect was blocked by flumazenil (1 mg/kg, i.p.), suggesting that apigenin modulates the benzodiazepine site of GABA_A_ receptors. Brown et al., [121] neuropharmacologically studied another flavone component chrysin. They administered chrysin (2 mg/kg, i.p.) or midazolam (1.5 mg/kg, i.p.) to rats 30 min before behavioral evaluations by an elevated plus maze test. Chrysin exerted an anxiolytic effect, which was affected by co-administered GABA_A_-benzodiazepine receptor antagonist flumazenil (3 mg/kg, i.p.).

Skullcap (*Scutellaria lateriflora*, Lamiaceae) is used as an herbal medicine for anxiety, sedation, insomnia and neuralgia. Awad et al., [122] measured the relative anxiety level of rats by an elevated plus maze test and confirmed the anxiolytic activity of an aqueous skullcap extract (1.0 mL of 100 mg extract/mL, p.o.). They identified two relevant flavone components as baicalein and baicalin (Figure 8). Based on the in vitro experimental result of baicalein that acted on the benzodiazepine binding site of GABA_A_ receptors, Liao et al., [123] verified in vivo effects of baicalein and its 7-glucuronide. Baicalein (10 mg/kg, i.p.) and baicalin (20 mg/kg, i.p.) induced anxiolysis in mice, which was antagonized by GABA_A_-benzodiazepine receptor antagonist flumazenil (2 mg/kg, i.p.).

Tea made from flavonoid-rich leaves of *Apocynum venetum* (Apocynaceae) is used in traditional Chinese medicine. Grundmann et al., [124] assessed the neurological activity of 70% ethanol extracts from the leaves of *Apocynum venetum* by an elevated plus maze test using mice. They demonstrated that the extracts are anxiolytic at two distinct concentrations of 22.5–30 mg/kg (p.o.) and 100–125 mg/kg (p.o.). They isolated flavonol kaempferol (Figure 8) from the active extracts. In their in vivo experiments, kaempferol (0.02–1.0 mg/kg, p.o.) showed an anxiolytic effect on mice with the potency comparable to that of diazepam (1.5 mg/kg, p.o.) and the effect of kaempferol (0.08 mg/kg, p.o.) was antagonized by GABA_A_-benzodiazepine receptor antagonist flumazenil (3 mg/kg, i.p.).

*Scutellaria baicalensis* (Lamiaceae) is a Chinese medicinal herb with the sedative and antibacterial property. Hui et al., [125] isolated flavone wogonin (Figure 8) from this plant. Their radioreceptor binding assay showed that wogonin has the affinity for the benzodiazepine site of GABA_A_ receptors in rat forebrain synaptosomal membranes with a *Ki* value of 0.92 µM. In the electrophysiological experiments, wogonin enhanced GABA-activated currents of rat dorsal root ganglion neurons and recombinant rat GABA_A_ receptors expressed in *Xenopus laevis* oocytes. Wogonin (7.5–30 mg/kg, p.o.) also showed in vivo anxiolytic effects on mice. Baicalein, baicalin and scutellarein (Figure 8) isolated from the root of *Scutellaria baicalensis* (Lamiaceae) bind to the benzodiazepine site of GABA_A_ receptors with lower affinity than that of wogonin [126].

The chalcone isoliquiritigenin (Figure 8) is contained in licorice, the root of *Glycyrrhiza glabra* (Fabaceae) that is used as a flavoring agent. Isoliquiritigenin was found to exert an anxiolytic effect on mice in an elevated plus maze test [127]. Cho et al., [128] demonstrated that isoliquiritigenin (25–50 mg/kg, p.o.) potentiates pentobarbital (45 mg/kg, i.p.)-induced sleep in mice, which was fully inhibited by GABA_A_-benzodiazepine receptor antagonist flumazenil (8 mg/kg, i.p.). Their radioreceptor binding assay and electrical measurement showed that isoliquiritigenin has the binding affinity for GABA_A_-benzodiazepine receptors in rat cerebral cortex membranes with a *Ki* value of 0.45 µM and it increases 2 µM GABA-evoked currents in rat dorsal raphe neurons by 151% at 10 µM. These results indicate that isoliquiritigenin is a positive allosteric modulator of GABA_A_-benzodiazepine receptors. Woo et al., [129] investigated the modulatory effect of isoliquiritigenin on GABAergic synaptic responses in mouse hippocampal CA1 pyramidal neurons by using a whole-cell patch clamp technique. Isoliquiritigenin prolonged at 1 µM the decay of spontaneous inhibitory postsynaptic currents mediated by GABA_A_ receptors. Its effect was inhibited by benzodiazepine antagonist flumazenil at 5 µM. Isoliquiritigenin also competitively binds to the glutamate recognition site of NMDA receptors in rat cerebral cortex membranes. Kawakami et al., [130] reported that isoliquiritigenin inhibited 300 µM NMDA-induced increase of the Ca^2+^ influx in cultured rat cortical neurons at 100–300 µM. Such an NMDA receptor antagonistic effect may contribute to protection against glutamate excitatory neurotoxicity rather than to production of anesthesia.

Cho et al., [131] revealed that ethanol extracts (250–500 mg/kg, p.o.) from licorice potentiate pentobarbital (45 mg/kg, i.p.)-induced sleep in mice, which was inhibited by benzodiazepine antagonist flumazenil (8 mg/kg, i.p.). Flavanone glabrol (Figure 8) isolated from the active extracts inhibited the binding of flumazenil to GABA_A_-benzodiazepine receptors in rat cerebral cortex membranes with the affinity of a *Ki* value of 1.63 µM. In in vivo experiments, glabrol (25–50 mg/kg, p.o.) increased the sleep duration and decreased the sleep latency of mice treated with pentobarbital (45 mg/kg, i.p.). These effects were blocked by flumazenil, suggesting that glabrol is a positive allosteric modulator of GABA_A_-benzodiazepine receptors.

The interactions of flavonoids with GABA_A_ receptors via multiple binding sites and their effects in the central nervous system were recently reviewed by Hanrahan et al., [132] and by Jäger and Saaby [133].

In addition to receptor and channel proteins, flavonoids mechanistically act on membrane lipids. Various flavonoids were reported to interact with phospholipid bilayers and biological membranes to modify their physicochemical properties [134,135,136]. Their membrane interactivity is also associated with antioxidant, antitumor and antibacterial effects [137].

## 6. Clinical Applicability of Phytochemicals

Previous in vitro and in vivo experiments indicate that phytochemical terpenoids, alkaloids and flavonoids exhibit the local and general anesthetic activity through the molecular mechanisms common to currently used anesthetics and anesthesia-related drugs. Different classes of phytochemicals to interact with receptors, ion channels and lipid membranes have been subjected to the administration experiments with animals and the pre-clinical trials in human subjects.

### 6.1. Local Anesthetic Phytochemicals

When administered to the conjunctival sac at μg/mL concentrations, linalool, linalyl acetate, menthol, α-terpineol, anethole, furanodiene-6-one, methoxyfuranoguaia-9-ene-8-one and (−)-β-caryophyllene induce local anesthesia in rats and rabbits, which reaches a maximum potency immediately after administration (within 5 min) and disappears in a short time (within 15 min) [30,33,51,53,54]. Despite promising experimental effects, however, these anesthetic terpenoids have not been applied to humans.

Although it is ideal for local anesthetics to block neuronal Na^+^ channels specifically, conventional local anesthetics are non-specific Na^+^ channel blockers that target not only Na^+^ but also K^+^ and Ca^2+^ channels. In contrast, neurotoxin alkaloids preferentially block Na^+^ channels by binding to the sites different from those of local anesthetics. Therefore, neurotoxin alkaloids acting on voltage-gated Na^+^ channels are expected to be lead compounds or alternatives for existing anesthetic agents [60]. Wang et al., [66] indicated the ability of bulleyaconitine A to potentiate infiltration anesthesia by in vitro and in vivo studies. Since bulleyaconitine A use-dependently blocked Nav1.7 and Nav1.8 Na^+^ currents in human embryonic kidney cells expressing neuronal Na^+^ channels, they subcutaneously injected bulleyaconitine A (≤125 μM) to rats in combination with 0.5% lidocaine and epinephrine (1:200,000). The duration of complete nociceptive blockade was increased from 3 to 24 h without adverse effects. When injecting test solutions in a volume of 0.2 mL to rat sciatic notch, 0.375 mM bulleyaconitine A induced sensory and motor blockade that completed 30–60 min after injection and started to recover 1.5–2 h after injection [65]. By co-injecting with 2% lidocaine or epinephrine (1:100,000), bulleyaconitine A prolonged the blocking time of both sensory and motor functions to 4 h with minimal systemic adverse effects. These results suggest that bulleyaconitine A readily diffuses through the nerve sheath and binds to Na^+^ channels with the relatively slow reversibility compared with conventional local anesthetics. The same local anesthetic property was found in 3-acetylaconitine, which has been used as an analgesic agent in China. Subcutaneous co-injection of 50–125 μM 3-acetylaconitine with 0.5% lidocaine and epinephrine (1:200,000) produced the complete cutaneous analgesia in rats that lasted for 3–12 h and the full recovery occurred ~5–8 days after injection [138]. Bulleyaconitine A and 3-acetylaconitine would be useful as an additive to prolong the effects of local anesthetics. Xu et al., [139] investigated the pharmacokinetics of bulleyaconitine A in rats after oral and intravenous administration. Bulleyaconitine A of 0.04, 0.12 and 0.36 mg/kg (p.o.) and 0.02 mg/kg (i.v.) showed mean *C*_max_ values of 2.11, 5.41, 11.47 and 19.97 ng/mL, respectively, and mean *T*_1/2_ values of 2.48, 1.93, 2.17 and 1.23 h, respectively. Weng et al., [140] performed a pharmacokinetic study of bulleyaconitine A in humans. Ten healthy male volunteers were intramuscularly injected with 0.2 mg bulleyaconitine A and blood plasma samples were collected before and 0.17–15 h after injection. Bulleyaconitine A showed a mean *C*_max_ value of 1.13 ng/mL and a mean *T*_max_ value of 0.9 h. However, eight of ten subjects claimed a distinct feeling of pain at the injection site, which started approximately at the time of the peak plasma concentrations and lasted 2–6 h.

Based on the finding that lappaconitine has the ability to block voltage-gated Na^+^ channels, Chen et al., [141] verified the effect of lappaconitine on humans by dividing 50 patients to be operated in the upper abdomen into five groups who received epidural injections of 0, 4, 8 and 12 mg lappaconitine or 2 mg morphine as a reference. Consequently, lappaconitine produced analgesia of the satisfactory potency and duration time but no side-effects like morphine, suggesting that the epidural injection of lappaconitine may be useful for postoperative analgesia. In their following study [142], 120 patients were divided into four groups who received epidural injections of test solutions consisting of 12 mg lappaconitine, 12 mg lappaconitine plus 22.5 mg bupivacaine, 22.5 mg bupivacaine or 2 mg morphine during the postoperative pain of incision operation. Epidural co-injection of lappaconitine with bupivacaine showed an analgesic effect with greater potency, earlier initiation and longer maintenance compared with lappaconitine alone and bupivacaine alone, indicating a benefit of lappaconitine co-administered with conventional local anesthetics.

Among Na^+^ channel-blocking *Aconitum* alkaloids, bulleyaconitine A in solution (i.m.), tablet (p.o.) and soft gel capsule (p.o.) have been prescribed for the treatment of chronic pain and rheumatoid arthritis in China since 1980s [66,140]. Lappaconitine preparations have been also manufactured in China. Long-lasting analgesia induced by bulleyaconitine A may be beneficial for postoperative pain control. Animal administration experiments, pre-clinical trials and pharmacokinetic studies have shown encouraging results. However, most aconitine-like alkaloids with the local anesthetic activity are known to have the drawback of a narrow therapeutic index and the possibility to cause hyperexcitability and cardiac arrhythmia [143].

### 6.2. General Anesthetic Phytochemicals

Plants of the genera *Passiflora*, *Valeriana*, *Matricaria*, *Scutellaria*, *Sideritis*, *Thymus*, *Scutellaria* and *Glycyrrhiz* have the property to modulate GABA_A_ receptor functions. Among them, several plant species of the genus *Passiflora* contain neuro-active alkaloids like harman and neuro-active flavonoids like apigenin, both of which have high affinity for GABA_A_ receptors. *Passiflora incarnata* extracts act on the benzodiazepine and ethanol binding sites of GABA_A_ receptors [82]. GABAergic phytochemicals in these plants are also expected to interact synergistically with benzodiazepines and barbiturates to potentiate their anesthetic, sedative and anxiolytic effects.

Movafegh et al., [144] verified the utility of *Passiflora incarnata* as a premedication by a double-blind and placebo-controlled study. Sixty outpatients undergoing inguinal herniorrhaphy were randomized into two groups who orally received either *Passiflora incarnata* extracts of 500 mg or placebo 90 min before surgery. When evaluating by a numeric rating scale before and 10–90 min after administration, the extracts reduced anxiety of patients without inducing sedation. Aslanargun et al., [145] performed a randomized, double-blind and placebo-controlled study to determine whether *Passiflora incarnata* is beneficial for patients undergoing anesthesia and surgery. Each 30 patients received orally commercially available aqueous extracts of *Passiflora incarnata* of 700 mg/5 mL or the same 5 mL-volume of placebo 30 min before spinal anesthesia, together with 3 mL of 0.5% bupivacaine. Their pre-clinical trial indicated the possibility of *Passiflora incarnata* premedication to suppress the increase of anxiety levels in the preoperative period without changing psychomotor functions.

Benzodiazepines are most widely prescribed for generalized anxiety disorder associated with the GABAergic system. Because of their adverse effects, however, much attention has been paid to an alternative agent. Akhondzadeh et al., [146] pre-clinically compared the efficacy of *Passiflora* extracts with benzodiazepine anxiolytics by a pilot double-blind randomized controlled trial. Eighteen outpatients orally received commercially available extracts of *Passiflora incarnata* 45 drops/day plus placebo tablet and other 18 outpatients orally received oxazepam 30 mg tablet/day plus placebo drops for four weeks. The extracts were proved to manage generalized anxiety disorder with the potency being almost comparable to that of oxazepam. The plant *Passiflora incarnata* has been listed as a safe herbal sedative by the US Food and Drug Administration [147]. Its extracts have been manufactured as drop (500 mg, Passipay™, Iran Darouk) [144] and syrup (700 mg/5 mL, Sandoz, Turkey) [145].

Chamomile, an herbal remedy for relaxation and calming, has been included in the pharmacopoeia of nearly 30 countries [148]. German chamomile *Matricaria recutita* (Asteraceae) is referred to as the most potent herb. Since in vitro and in vivo animal experiments indicated that chamomile and its flavone component apigenin exert neuro-modulatory effects by acting on the benzodiazepine site of GABA_A_ receptors, Amsterdam et al., [149] conducted a randomized, double-blind, placebo-controlled trial of the chamomile therapy for humans with generalized anxiety disorder. Fifty-seven outpatients were randomized into two groups to receive orally either double-blind chamomile extracts or placebos for eight weeks. Chamomile samples were prepared as 220 mg capsules containing the pharmaceutical grade German chamomile extract standardized to a content of 1.2% apigenin. Anxiety rating scores showed clinically meaningful difference between two groups, suggesting an anxiolytic effect of chamomile that is associated with GABA_A_ receptor-acting apigenin.

Skullcap *Scutellaria lateriflora* (Lamiaceae) is a medicinal herb that has been traditionally used for anxiety, sedation and neuralgia. Wolfson and Hoffmann [150] performed a double-blind and placebo-controlled study, in which 19 healthy volunteers orally received one 350-mg capsule of freeze-dried skullcap, one 100-mg capsule of skullcap extract or two 100-mg capsules of skullcap extract. They found the significant anxiolytic activity of all skullcap preparations. Skullcap contains wogonin, baicalein, baicalin and scutellarein [151]. These flavones are considered to exert neuropharmacological effects by binding to the benzodiazepine site of GABA_A_ receptors.

### 6.3. Methodological Considerations in Discovering Phytochemical Lead Compounds

If the anesthetic activity is found in plant samples by screening or preliminary experiments, they are subjected to specification of the relevant phytochemical components. Fractionation and isolation of active substances are commonly guided by the assays of bioactivity and affinity to pharmacological targets. Local anesthetic terpenoids were purified from *Commiphora molmol* (Burseraceae) by bioassay-guided chromatography [53]. Anxiolytic flavonol was isolated from *Apocynum venetum* (Apocynaceae) by chromatographic fractionation combined with an elevated plus maze test [124]. The mode of action on anesthetic-targeting channels and receptors is usable as an isolation index. Anesthetic flavonoids were isolated from *Tanacetum parthenium* (Asteraceae) [115], *Valeriana wallichii* (Valerianaceae) [116] and *Artemisia herba-alba* (Asteraceae) [119] by preparative chromatography that was guided by the benzodiazepine radioligand binding assay with GABA_A_ receptors. Phytochemicals mechanistically interact with neuronal membranes as well as anesthetics and anesthesia-related drugs [11,152,153,154]. In addition to bioassay and radioligand binding assay, the membrane interactivity would give another clue to discover anesthetic phytochemicals.

Even if in vivo applications and pre-clinical trials of plant preparations offered the positive evidence for anesthetic effects on experimental animals and human subjects, caution should be taken to interpret such results. The researches to find drug candidates in plants have generally focused on a single bioactive entity. However, attempts to purify and isolate the relevant phytochemicals may be self-defeating because the overall pharmacological activity of plants can rely on the synergistic interaction between different components [155]. A whole extract or a partially purified extract from plants could have advantages over a single isolated phytochemical as suggested by the phenomenon that a combination of some plants are more effective than either plant alone.

## 7. Conclusions

Results of the literature search suggest that plant preparations and their containing phytochemicals have the potential to become local anesthetic, general anesthetic, antinociceptive, analgesic or sedative drugs. However, well-controlled clinical trials with phytochemical drug candidates and their practical applications to humans are still limited. Nevertheless, there is a possibility that selected phytochemicals could lead to anesthetics and anesthesia-related drugs. Terpenoids, alkaloids and flavonoids are expected to become novel anesthetic agents of plant origin because they meet the mechanistic requirements to interact with receptors, channels and membranes and they have the characteristic molecular structures different from conventional drugs.

Phytochemicals with the anesthetic activity are summarized in Table 1. Among them, voltage-gated Na^+^ channel-blocking alkaloids (such as lappaconitine, bulleyaconitine A, 3-acetylaconitine, etc.) and terpenoids (such as menthol, thymol, carvacrol, linalool, etc.) may be the alternatives for local anesthetics. Their satisfactory results have been obtained from in vitro and in vivo experiments with animals and pre-clinical trials in human subjects. GABA_A_ receptor-modulatory flavonoids (such as apigenin, kaempferol, baicalein, wogonin, chrysin, isoliquiritigenin, etc.) may be the lead compounds for general anesthetics and sedatives. Some of them have been successfully applied to experimental animals and humans. Clinical efficacy, adverse action, pharmacokinetics and pharmacodynamics of these phytochemicals remain to be elucidated together with structural modification to enhance the activity or reduce the toxicity.

## Figures and Tables

**Table 1:** Phytochemicals with anesthetic activity.

Phytochemicals	Activities	Experiments	Results	References
Terpenoids				
Linalool	LA	In vitro: Monitoring of compound action potentials in frog sciatic nerves	Exerted local anesthetic effects at 7.5–30 mM as well as 3.5–30 mM lidocaine	[29]
Linalool Linalyl acetate	LA	In vivo: Administration in rabbit conjunctival sac	Depressed conjunctival reflexes by 0.03–2.5 mg/mL administration	[30]
Linalool	LA	In vitro: Patch-clamp recording of rat sciatic nerves and rat dorsal root ganglion neurons	Reversely blocked nerve excitability and inhibited voltage-gated Na^+^ currents at sub-micromolar concentrations	[31]
(−)-Menthol (+)-Menthol	LA	In vitro and in vivo: Evaluation of the activity using rat phrenic nerve hemidiaphragm and by rabbit conjunctival reflex test	Reduced the electrically evoked contractions at 0.1–100 ng/mL and increased the number of stimuli to provoke the conjunctival reflex at 30–300 μg/mL	[33]
Menthol	LA	In vitro: Patch-clamp recording of rat dorsal root ganglion neurons	Inhibited tetrodotoxin-resistant Nav1.8 and Nav1.9 and tetrodotoxin-sensitive Na^+^ channels depending on micromolar concentration, voltage and frequency	[34]
Carvacrol Thymol Citronellol Bornyl acetate Citral	LA	In vitro: Recording of compound action potentials in frog sciatic nerves	Reduced compound action potential peak amplitudes (IC_50_ = 0.34–7.2 mM) and inhibited nerve conduction by blocking tetrodotoxin-sensitive voltage-gated Na^+^ channels	[36,37,38]
Carvacrol	LA	In vitro: Monitoring of rat sciatic nerve compound action potentials and patch-clamp recording of rat dorsal root ganglion neurons	Reversely blocked the excitability of sciatic nerves (IC_50_ = 0.5 mM) and reduced voltage-gated Na^+^ currents (IC_50_ = 0.37 mM)	[42]
Carvacrol	AA LA	In vivo: 50–100 mg/kg (p.o.) administration to mice, followed by acetic acid-induced abdominal constriction, formalin injection and hot plate tests	Inhibited nociception induced by different methods	[43]
(−)-Carvone	AA LA	In vivo: 100–200 mg/kg (i.p.) administration to mice	Inhibited acetic acid-induced writhing and formalin-induced hind paw nociception	[44]
Estragole	LA	In vitro: Patch-clamp recording of rat dorsal root ganglion neurons	Inhibited total Na^+^ currents (IC_50_ = 3.2 mM) and tetrodotoxin-resistant Na^+^ currents (IC_50_ = 3.6 mM)	[46]
Citral	LA	In vitro: Monitoring of compound action potentials in rat sciatic nerves	Inhibited compound action potentials (IC_50_ = 0.23 mM)	[48]
α-Terpineol Anethole	LA	In vitro and in vivo: Evaluation of the activity using rat phrenic nerve-hemidiaphragm and by rabbit conjunctival reflex test	Reduced the electrically evoked contractions at 0.01–1 μg/mL and increased the number of stimuli to evoke the conjunctival reflex at 10–100 μg/mL	[51]
*p*-Cymene	AA LA	In vivo: 50–200 mg/kg (i.p.) administration to mice, followed by acetic acid-induced writhing and formalin-induced hind paw licking tests	Showed significant antinociceptive effects in both tests	[52]
β-Caryophyllene	LA	In vitro and in vivo: Evaluation of the activity using rat phrenic nerve-hemidiaphragm and by rabbit conjunctival reflex test	Reduced the electrically evoked contractions at 0.1 ng/mL to 1.0 μg/mL and increased the number of stimuli to evoke the conjunctival reflex at 10 μg/mL to 1.0 mg/mL	[54]
Alkaloids				
Lappaconitine other neurotoxins	LA	In vivo: Rabbit corneal reflex test to drop test solutions (0.01–1%) into the conjunctival sac and cat neck trunk anesthesia to inject test solutions (0.1 mL of 0.1–0.5%, i.c. and s.c.)	Showed greater potency and longer duration of anesthesia than lidocaine and procaine	[61]
Aconitine 3-Acetylaconitine	AA	In vivo: Intravenous administration to mice, followed by formaldehyde injection (s.c.) to induce hyperalgesia	Showed antinociceptive effects in the early phase (ED_50_ = 0.027–0.028 mg/kg) and the late phase (ED_50_ = 0.077–0.097 mg/kg)	[63]
Bulleyaconitine A	LA	In vivo: Injection of 0.2 mL test solution into rat sciatic notch	Blocked sensory and motor functions of the sciatic nerves at 0.375 mM Co-injection with 2% lidocaine or epinephrine (1:100,000) prolonged the nerve-blocking duration	[65]
Bulleyaconitine A	LA AA	In vitro and in vivo: Recording of Na^+^ currents of human embryonic kidney cells expressing Nav isoforms and measurement of the cutaneous trunci muscle reflex after injection (s.c.) of 0.6 mL test solution	Blocked Nav1.7 and Nav1.8 Na^+^ currents at 10 μM and induced the complete nociceptive blockade lasting for ~3 h at 0.125 mM Co-injection with 0.5% lidocaine/epinephrine (1:200,000) increased the duration of analgesia	[66]
3-Acetylaconitine	AA LA	In vivo: 50–125 μM (s.c.) co-injection with 0.5% lidocaine and epinephrine (1:200,000) to rats	Produced the complete analgesia lasting for 3–12 h	[138]
Lappaconitine	AA LA	Pre-clinical: Patients received epidural injections of 0–12 mg lappaconitine	Produced satisfactory analgesia depending on dosages Suggested the clinical utility for postoperative analgesia	[141]
Lappaconitine	AA LA	Pre-clinical: Patients received epidural injections of test solutions consisting of 12 mg lappaconitine, 12 mg lappaconitine plus 22.5 mg bupivacaine or 22.5 mg bupivacaine	Epidural co-injection of lappaconitine with bupivacaine induced analgesia with greater potency, earlier initiation and longer maintenance than lappaconitine alone and bupivacaine alone	[142]
Flavonoids				
(−)-Epigallo-catechin-3-gallate	LA	In vitro: Measurement of Na^+^ currents in rat dorsal root ganglion neurons	Inhibited tetrodotoxin-sensitive and tetrodotoxin-resistant Na^+^ currents	[71]
Genistein Daidzein	LA	In vitro: Measurement of Na^+^ currents in cultured rat brain neurons	Blocked voltage-sensitive Na^+^ channels (IC_50_ = 60 μM for genistein and 195 μM for daidzein)	[72]
Stilbenoid				
Resveratrol	LA	In vitro: Measurement of Na^+^ currents in rat dorsal root ganglion neurons	Suppressed tetrodotoxin-sensitive and tetrodotoxin-resistant Na^+^ channels	[75]
Terpenoids				
Aristolen-1(10)-en-9-ol	S H GA-like	In vivo: Spontaneous vapor administration to mice	Inhibited the locomotion of caffeine-treated mice at 300 μg/cage and prolonged the time of pentobarbital-induced sleep The effects were inhibited by flumazenil	[89]
Isopulegol Pinocarveol Verbenol Myrtenol	GA-like	In vitro: Determination of the effects on GABA_A_ receptor α1β2 or α1β2γ2 subunits expressed in *Xenopus laevis* oocytes or human embryonic kidney cells	Modulated GABA_A_ receptor functions at 3–300 μM independently of the γ2 subunit	[92]
Valerenic acid	GA-like	In vitro: Determination of the effect on GABA_A_ receptors in neonatal rat brainstem preparations	Inhibited muscimol-sensitive neurons (IC_50_ = 23 μM) The effect was antagonized by bicuculline	[93]
Valerenic acid	GA-like A	In vitro and in vivo: Radioligand binding assay with crude rat brain membranes and 1–6 mg/kg (i.p.) and 10 mg/kg (p.o.) administration to mice	Interacted allosterically with benzodiazepine and GABA binding site of GABA_A_ receptors Showed anxiolytic effects on mice	[96]
Linalool	S H GA-like	In vivo: Placed mice in a chamber of an atomosphere saturated 1% or 3% vapor	Produced sedation and increased the time of pentobarbital-induced sleep	[99]
Menthol	GA-like	In vitro: Determination of the effect on recombinant human GABA_A_ receptors expressed in *Xenopus laevis* oocytes	Enhanced sub-maximal GABA currents at 3–300 μM	[102]
Menthol	GA-like	In vitro: Whole-cell voltage clamp recording of rat midbrain periaqueductal grey neurons	Prolonged at 150–750 μM the duration of spontaneous inhibitory postsynaptic potentials mediated by GABA_A_ receptors	[103]
Thymol Carvacrol Eugenol	GA-like	In vitro: Determination of the effects on primary cultures of mouse cortical neurons at micromolar concentrations	Enhanced [^3^H]flunitrazepam binding to GABA_A_ receptors and increased 2 μM GABA-evoked Cl^−^ influx	[15]
Thymol	GA-like	In vitro: Determination of the effects on human GABA_A_ receptor subunit combinations expressed in *Xenopus laevis* oocytes	Potentiated EC_20_ GABA response at 1–100 μM	[104]
Thymol	GA-like	In vitro: Determination of the positive allosteric modulatory effects on GABA_A_ receptors in primary cultures of mouse cortical neurons	Enhanced 5 μM GABA-induced Cl^−^ influx (EC_50_ = 12 μM) and directly enhanced Cl^−^ influx (EC_50_ = 135 μM)	[14]
Alkaloids				
Harman	A S	In vivo: 2.5–10 mg/kg (i.p.) administration to rats	Decreased the time of immobility in a forced swim test and increased the time spent in open arms in an elevated plus maze	[110]
Harman Norharman Harmine	S GA-like	In vivo: 2.5–15 mg/kg (i.p.) administration to mice	Decreased the time of immobility The effects were inhibited by flumazenil	[111]
Flavonoids				
(−)-Epigallo-catechin-3-gallate	H	In vivo: 5–20 mg/kg (p.o.) administration to mice	Prolonged the time of pentobarbital-induced sleep	[117]
Apigenin	S GA-like	In vivo: 0.6 mg/kg (p.o.) administration to mice	Induced sedation The effect was blocked by flumazenil	[120]
Chrysin	A GA-like	In vivo: 2 mg/kg (i.p.) administration to rats	Induced anxiolysis The effect was affected by flumazenil	[121]
Baicalein Baicalin	A GA-like	In vivo: 10–20 mg/kg (i.p.) administration to mice	Induced anxiolysis The effects were antagonized by flumazenil	[123]
Kaempferol	A GA-like	In vivo: 0.02–1.0 mg/kg (p.o.) administration to mice	Induced anxiolysis The effect was antagonized by flumazenil	[124]
Wogonin	GA-like A	In vitro and in vivo: Radioreceptor binding assay with rat forebrain synaptosomal membranes, electrophysiological experiment with rat dorsal root ganglion neurons and 7.5–30 mg/kg (p.o.) administration to mice	Showed the affinity for the benzodiazepine site of GABA_A_ receptors (*Ki* = 0.92 μM), enhanced GABA-activated currents and induced anxiolysis	[125]
Isoliquiritigenin	GA-like H	In vitro and in vivo: Radioreceptor binding assay with rat cerebral cortex membranes, electrical measurement of rat dorsal raphe neurons and 25–50 mg/kg (p.o.) administration to mice	Showed the affinity for GABA_A_-benzodiazepine receptors (*Ki* = 0.45 μM), increased GABA-evoked currents and potentiated pentobarbital-induced sleep	[128]
Isoliquiritigenin	GA-like	In vitro: Measurement of the GABAergic synaptic renponses by the whole-cell patch clamp technique	Prolonged at 1 μM the decay of spontaneous inhibitory postsynaptic currents mediated by GABA_A_ receptors The effect was inhibited by flumazenil	[129]
Glabrol	H GA-like	In vivo: 25–50 mg/kg (p.o.) administration to mice	Increased the sleep duration and decreased the sleep latency in mice treated with pentobarbital The effects were blocked by flumazenil	[131]
Apigenin	A	Pre-clinical: Randomized, double-blind, placebo-controlled trial for outpatients with 220 mg chamomile extracts standardized to a content of 1.2% apigenin	Showed clinically meaningful difference from controls in anxiety rating scores	[149]
Wogonin Baicalein Baicalin Scutellarein	A	Pre-clinical: Double-blind and placebo-controlled study for human subjects with 100–200 mg skullcap extracts containing flavones	Exhibited anxiolytic effects	[150,151]

LA: local anesthetic; GA: general anesthetic; AA: antinociceptive/analgesic; S: sedative; A: anxiolytic; H: hypnotic.

## Abbreviations

The following abbreviations are used in this manuscript:

CAP	Compound action potential
GABA_A_ receptor	γ-aminobutyric acid type A receptor
Nav channel	Voltage-gated Na^+^ channel
NMDA receptor	*N*-methyl-d-aspartate receptor
TTX	Tetrodotoxin
